# Continuous regional arterial infusion for acute pancreatitis: a propensity score analysis using a nationwide administrative database

**DOI:** 10.1186/cc13029

**Published:** 2013-10-02

**Authors:** Tsuyoshi Hamada, Hideo Yasunaga, Yousuke Nakai, Hiroyuki Isayama, Hiromasa Horiguchi, Shinya Matsuda, Kiyohide Fushimi, Kazuhiko Koike

**Affiliations:** 1 Department of Gastroenterology, Graduate School of Medicine, The University of Tokyo, 7-3-1 Hongo, Bunkyo-ku, Tokyo 113-8655, Japan; 2 Department of Health Economics and Epidemiology Research, School of Public Health, The University of Tokyo, 7-3-1 Hongo, Bunkyo-ku, Tokyo 113-8655, Japan; 3 Department of Clinical Data Management and Research, Clinical Research Center, National Hospital Organization Headquarters, 2-5-21 Higashigaoka, Meguro-ku, Tokyo 152-8621, Japan; 4 Department of Preventive Medicine and Community Health, University of Occupational and Environmental Health, 1-1 Iseigaoka, Yahatanishi-ku, Kitakyushu 807-8555, Japan; 5 Department of Health Care Informatics, Tokyo Medical and Dental University, 1-5-45 Yushima, Bunkyo-ku, Tokyo 113-8510, Japan

## Abstract

**Introduction:**

Although continuous regional arterial infusion (CRAI) of a protease inhibitor and an antibiotic may be effective in patients with severe acute pancreatitis, CRAI has not yet been validated in large patient populations. We therefore evaluated the effectiveness of CRAI based on data from a national administrative database covering 1,032 Japanese hospitals.

**Methods:**

In-hospital mortality, length of stay and costs were compared in the CRAI and non-CRAI groups, using propensity score analysis to adjust for treatment selection bias.

**Results:**

A total of 17,415 eligible patients with acute pancreatitis were identified between 1 July and 30 September 2011, including 287 (1.6%) patients who underwent CRAI. One-to-one propensity-score matching generated 207 pairs with well-balanced baseline characteristics. In-hospital mortality rates were similar in the CRAI and non-CRAI groups (7.7% vs. 8.7%; odds ratio, 0.88; 95% confidence interval, 0.44–1.78, *P* = 0.720). CRAI was associated with significantly longer median hospital stay (29 vs. 18 days, *P* < 0.001), significantly higher median total cost (21,800 vs. 12,600 United States dollars, *P* < 0.001), and a higher rate of interventions for infectious complications, such as endoscopic/surgical necrosectomy or percutaneous drainage (2.9% vs. 0.5%, *P* = 0.061).

**Conclusions:**

CRAI was not effective in reducing in-hospital mortality rate in patients with acute pancreatitis, but was associated with longer hospital stay and higher costs. Randomized controlled trials in large numbers of patients are required to further evaluate CRAI for this indication.

## Introduction

Acute pancreatitis is an inflammatory disease of the pancreas with a wide spectrum of severity. It is characterized by autodigestion of the pancreas due to activation of inherent proteases and frequently involves surrounding retroperitoneal tissues and/or remote organ systems. Although recent advances in intensive care and aggressive treatment methods specialized for acute pancreatitis [1,2] have dramatically reduced the mortality rate associated with this condition, mortality due to severe necrotizing pancreatitis is not uncommon [3,4]. Protease inhibitors may directly suppress pancreatic inflammation by inhibiting pancreatic enzymes and improving coagulopathy [5,6]. In addition, antibiotics have been reported to reduce the mortality associated with severe acute pancreatitis by preventing secondary infection of the necrotic pancreatic tissue [7,8], although its effectiveness on mild acute pancreatitis has not been demonstrated [3]. In acute necrotizing pancreatitis, however, pancreatic ischemia due to vasospasm and enhanced intravascular coagulation inhibit protease inhibitors and prevent intravenously administered antibiotics from penetrating into pancreatic tissue.

Continuous regional arterial infusion (CRAI), rather than systemic intravenous administration, may be a theoretically reasonable drug delivery system for patients with severe acute pancreatitis. Administration of a protease inhibitor through a catheter placed into one of the arteries supplying the inflamed pancreas can dramatically increase the effective concentration of this agent in the pancreatic parenchyma [9,10]. To date, several retrospective, uncontrolled case series have suggested that CRAI may reduce mortality associated with acute necrotizing pancreatitis [11-15]. To the best of our knowledge, however, only one randomized controlled trial has assessed mortality rates in patients administered a protease inhibitor and an antibiotic via CRAI [16]. In that study, which involved 78 patients with severe acute pancreatitis, the mortality rate was significantly lower in the CRAI group than in the non-CRAI group (5.1% vs. 23.1%; *P* = 0.02). Owing to the small sample size and lack of stratification, however, the characteristics of patients in the CRAI and non-CRAI groups were poorly balanced, with the CRAI group being younger and having a better computed tomography (CT) severity index, possibly leading to an overestimation of the efficacy of CRAI [17]. Thus, the effectiveness of CRAI for acute necrotizing pancreatitis remains unclear.

We therefore evaluated the effectiveness of CRAI of a protease inhibitor and an antibiotic in patients with acute pancreatitis by performing a one-to-one propensity score–matched analysis of patients with or without CRAI derived from a nationwide administrative database of inpatient care in Japan.

## Materials and methods

### Data source

The Diagnosis Procedure Combination (DPC) database is an original case mix administrative database of inpatients in Japan that provides data on admission and discharge abstracts as well as administrative claims. The DPC system has been adopted by acute care hospitals in Japan [18,19]. Data on approximately seven million inpatients hospitalized between 1 July 2010 and 30 June 2011 were collected from more than 1,000 hospitals throughout Japan, representing approximately 50% of all acute care hospitalizations during the same period in Japan. The main diagnoses, comorbidities present at admission and complications during hospitalization were recorded using the *International Classification of Diseases and Related Health Problems, 10th Revision* (ICD-10) codes and text data in the Japanese language. The database also contains detailed medical information, such as patients’ age and sex; length of hospital stay; discharge status, including in-hospital death; unique identifiers of the hospitals; types of hospitals (academic and nonacademic); interventional and surgical procedures; medications and devices indexed by the original codes in Japanese; and cost data. The database also includes consciousness status at the time of admission and discharge based on the Japan Coma Scale (JCS), in which a score of 0 indicates alert consciousness, scores of 1 to 3 indicate wakefulness without any stimuli, scores of 10 to 30 indicate arousal by some stimuli and scores of 100 to 300 indicate coma. The JCS and the Glasgow Coma Scale assessments are well-correlated [20,21]. Each patient with a principal diagnosis of acute pancreatitis was given the highest prognostic factor and CT severity index scores within 48 hours of admission according to the Japanese severity scoring system by the attending physicians [22,23].

This study was approved by the institutional review board of The University of Tokyo Hospital, which waived the requirement for patient informed consent because of the anonymous nature of the data.

### Japanese severity scoring system for acute pancreatitis

The severity of acute pancreatitis was determined for each patient on the basis of the Japanese severity score (prognostic factor score) determined by summing nine factors, along with the CT severity score [22,24]. Table 1 shows the details of this scoring system. Severe acute pancreatitis was diagnosed when the total prognostic factor score was 3 or higher or the CT severity grade was 2 or higher [22].

**Table 1:** **Japanese severity scoring system for acute pancreatitis of the Ministry of Health, Labour and Welfare of Japan (2008 revision)**^**a**^

**Scoring system**	**Basis for score**
Prognostic factor score (one point for each factor)	
1	Base excess less than or equal to -3 mEq/L or shock (systolic blood pressure below 80 mmHg)
2	PaO_2_ ≤60 mmHg (room air) or respiratory failure (respiratory assistance needed)
3	BUN ≥40 mg/dl or (or creatinine ≥2.0 mg/dl) or oliguria (daily urine output <400 ml even after intravenous fluid resuscitation)
4	LDH at or above twice the upper limit of normal
5	Platelet count ≤100,000/mm^3^
6	Serum calcium ≤7.5 mg/dl
7	CRP ≥15 mg/dl
8	Number of positive measures in SIRS criteria ≥3
9	Age ≥70 years
CT grade based on contrast-enhanced CT	
1	Extrapancreatic progression of inflammation
	Anterior pararenal space, zero points
	Root of mesocolon, one point
	Beyond lower pole of kidney, two points
2	Hypoenhanced lesion of the pancreas
	Pancreas conveniently divided into three segments (head, body and tail).
	Localized in each segment or surrounding only the pancreas, zero points
	Extends to two segments, one point
	Occupies two whole segments or more, two points
	Factors 1 + 2 = total score
	Total score = 0 or 1, grade 1
	Total score = 2, grade 2
	Total score = 3 or more, grade 3
Assessment of severity	If prognostic factor score is ≥3 or CT grade is ≥2, acute pancreatitis is considered severe.

^a^BUN, Blood urea nitrogen; CRP, C-reactive protein; CT, Computed tomography; LDH, Lactate dehydrogenase; PaO_2_, partial pressure of oxygen in blood; SIRS, Systemic inflammatory response syndrome. Measures within the SIRS criteria include body temperature above 38°C or less than 36°C, heart rate more than 90 beats/min, respiratory rate more than 20 breaths/min or partial pressure of carbon dioxide in blood less than 32 torr, as well as white blood cell count above 12,000 cells/mm^3^, less than 4,000 cells/mm^3 ^or greater than 10% immature (band) forms. The 2008 revision of the Japanese severity scoring system is contained in [22].

### Continuous regional arterial infusion of a protease inhibitor and an antibiotic

CRAI consisted of the continuous infusion of a protease inhibitor and an antibiotic (usually carbapenem) through a catheter inserted into one of the arteries perfusing the inflamed lesion of the pancreas. The general methods of CRAI were as follows. The catheter used for CRAI was the same as the one used for angiography. Following CT evaluation of the hypoenhanced area of the pancreas, angiography of the pancreas was performed. The catheter tip was located in the artery perfusing the area containing the main lesion of hypoperfusion of the pancreas. If the main lesion was located in the head of the pancreas, the catheter tip was placed in the common hepatic, gastroduodenal or superior mesenteric artery; if the main lesion was located in the body or tail of the pancreas, the catheter tip was placed in the celiac, splenic or dorsal pancreatic artery; and if the lesion involved the entire pancreas, the catheter tip was placed in the celiac artery. CRAI was usually started within 2 or 3 days of admission.

### Patient selection and data

We identified all adult patients (20 years of age or older) who were admitted to the participating hospitals with a principal diagnosis of acute pancreatitis (ICD-10 code K85) and were discharged between 1 July 2010 and 30 September 2011. Patients transferred within 7 days of hospitalization were excluded because transfer was based on Japanese guidelines recommending that patients with a prognostic factor score of 3 or higher be transferred to a specialized medical institution [22]. Patients who underwent CRAI for acute pancreatitis were identified based on the performance of selective arterial angiography combined with the infusion of a protease inhibitor (gabexate mesilate or nafamostat mesilate) and a carbapenem antibiotic (meropenem, imipenem, doripenem, biapenem or panipenem). The cohort for propensity score analysis was generated by combining those patients who underwent CRAI within 3 days of admission and patients who had intravenous infusions with both a protease inhibitor and a carbapenem antibiotic within 3 days of admission. The types of protease inhibitors and antibiotics initially administered via CRAI were also recorded.

The baseline characteristics, prognostic factor scores and CT severity scores at the time of admission of each patient were recorded. JCS [20,21] was categorized into four groups: 0, 1 to 3, 10 to 30 and 100 to 300. Comorbidities, assessed by recording ICD-10 codes, were converted into scores, and these scores were summed to calculate the Charlson comorbidity index (CCI) score for each patient based on Quan’s algorithm [25]. Hospital volume was defined as the number of inpatients with acute pancreatitis per year per hospital and was categorized into quartiles (very low, low, high and very high volume) containing approximately equal numbers of patients [26]. Hospital type was categorized as academic or nonacademic.

### Outcomes

The primary outcome of this study was in-hospital mortality. Secondary outcomes included length of hospital stay, total costs and requirement of intervention for infectious complications, that is, endoscopic or surgical necrosectomy or percutaneous drainage.

### Statistical analyses

A retrospective observational design was utilized to evaluate the effectiveness of CRAI of a protease inhibitor and an antibiotic for acute pancreatitis. Because CRAI is likely to be performed in more severely ill patients, an unadjusted comparison of CRAI and non-CRAI groups may be subject to treatment selection bias [27,28], resulting in an underestimation of the effectiveness of CRAI. To overcome this bias, we performed a propensity score analysis [27,28]. A propensity score was calculated for each patient in the cohort as described, using a logistic regression model with baseline variables that potentially influenced the selection of CRAI, including age, sex, JCS score, CCI score, prognostic factor score, CT severity index score, hospital volume and hospital type. We then matched patients in the CRAI patient cohort one-to-one with patients in the non-CRAI group by propensity score matching using the estimated propensity scores of each patient based on the nearest neighbor method within a caliper. In this algorithm, each patient in the non-CRAI group was matched with a patient in the CRAI group with the closest estimated propensity score within a specified range (≤0.25 of the pooled standard deviation of estimated propensity scores) [29]. C-statistics were used to evaluate goodness of fit.

Continuous variables were described as means and standard deviations or medians and interquartile ranges (IQRs) and were compared using Student’s *t-*test or the Mann–Whitney *U* test as appropriate. Between-group differences in categorical variables were compared using Fisher’s exact test or a χ^2^ test as appropriate. The trend toward higher in-hospital mortality with later administration of CRAI was evaluated using the Cochrane-Armitage trend test. A multivariate logistic regression model was used to compare in-hospital mortality in the CRAI and non-CRAI groups, with adjustment of propensity score quintiles.

The Cochrane-Armitage trend test was performed using R software version 2.15.1 (R Development Core Team; http://www.r-project.org). All other statistical analyses were performed using IBM SPSS Statistics version 19 software (IBM, Armonk, NY, USA). *P* < 0.05 was considered statistically significant.

## Results

### Patient selection and matching

During the 15-month study period, 21,468 patients ages 20 years and older were hospitalized with acute pancreatitis at the 1,032 DPC participating hospitals (116 academic and 916 nonacademic hospitals). We identified 17,415 patients whose prognostic factor scores and CT severity scores upon admission were both recorded and who had not been transferred within 7 days of hospitalization. From among these 17,415 patients, 287 (1.6%) underwent CRAI at 29 academic and 70 nonacademic hospitals. The cohort used for propensity score analysis consisted of 247 patients who underwent CRAI within 3 days of admission (the CRAI group) and 1,307 patients who underwent intravenous administration of a protease inhibitor and a carbapenem antibiotic within 3 days of admission (the non-CRAI group).

**Figure 1:**
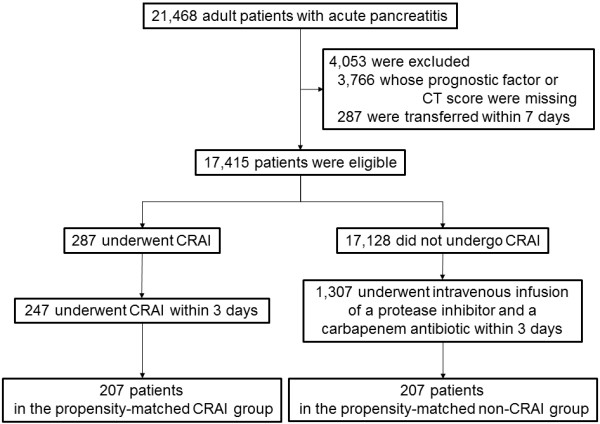
**Flowchart of patients with acute pancreatitis and propensity matching of patients with or without continuous regional arterial infusion.** CRAI, Continuous regional arterial infusion; CT, Computed tomography.

Patients in the CRAI and non-CRAI groups had mean propensity scores of 0.334 (95% confidence interval (CI) = 0.308 to 0.360) and 0.126 (95% CI = 0.119 to 0.133), respectively. The C-statistic (area under the receiver operating characteristic curve) was 0.811. Using the algorithm described above, we were able to match 207 patients in the CRAI group with 207 in the non-CRAI group (Figure 1).

### Patient characteristics

The characteristics of all patients in the CRAI and non-CRAI groups (*n* = 1,554) and in the 207 propensity-matched pairs (*n* = 414) are shown in Table 2. Analysis of the total patient population showed that patients in the CRAI group was younger and more likely to be treated in academic hospitals, and they had higher prognostic factor and CT severity index scores. CRAI was administered on day 1 of admission to 109 patients (52.7%), on day 2 to 60 patients (29.0%), on day 3 to 31 patients (15.0%) and on day 4 to 7 patients (3.4%).

**Table 2:** **Characteristics of patients in the unmatched and propensity-matched groups with or without continuous regional arterial infusion**^**a**^

	**Unmatched groups**		***P***	**Propensity-matched groups**		***P***
**Patient characteristics**	**CRAI ** **(*n*** **= 247)**	**Non-CRAI ** **(*n*** **= 1,307)**		**CRAI ** **(*n*** **= 207)**	**Non-CRAI ** **(*n*** **= 207)**	
Mean age (years)	54.6 ± 16.4	60.6 ± 18.1	<0.001	56.7 ± 16.3	56.0 ± 17.6	0.684
Sex						
Males	69.2%	67.6%	0.622	69.6%	66.7%	0.527
Females	30.8%	32.4%		30.4%	33.3%	
CCI score						
0	47.4%	43.1%	0.404	48.8%	48.3%	0.944
1 or 2	41.7%	46.2%		42.5%	42.0%	
≥ 3	10.9%	10.7%		8.7%	9.7%	
JCS score						
0	89.9%	94.3%	0.407	90.8%	92.3%	0.711
1 to 3	6.9%	4.5%		6.8%	5.8%	
10 to 30	2.0%	0.8%		1.4%	0.5%	
100 to 300	1.2%	0.4%		1.0%	1.4%	
Prognostic factor score						
0	29.1%	48.3%	<0.001	34.3%	35.3%	0.970
1	12.6%	24.3%		14.0%	14.5%	
2	17.4%	11.1%		18.4%	15.0%	
3	20.2%	7.8%		16.9%	16.9%	
4	12.1%	3.6%		8.2%	8.2%	
5	2.4%	2.3%		2.4%	2.4%	
≥ 6	6.1%	2.7%		5.8%	7.7%	
CT severity score						
0	13.4%	47.8%	<0.001	15.9%	15.5%	0.999
1	9.3%	20.4%		11.1%	10.6%	
2	38.9%	21.7%		42.0%	43.0%	
3	23.9%	6.0%		18.4%	18.8%	
4	14.6%	4.1%		12.6%	12.1%	
Hospital volume (per 12 months)						
≤ 14	21.5%	20.0%	0.324	21.7%	24.6%	0.835
15 to 22	21.1%	26.3%		22.2%	23.7%	
23 to 32	24.3%	24.3%		23.7%	21.3%	
≥ 33	33.2%	29.4%		32.4%	30.4%	
Hospital type						
Academic	31.2%	21.3%	0.001	28.0%	24.6%	0.435
Community	68.8%	78.7%		72.0%	75.4%	

^a^Age shown as mean and standard deviation. Prognostic factor score and CT severity score were based on the Japanese severity scoring system for acute pancreatitis (2008 revision) [22]. CCI, Charlson comorbidity index; CRAI, Continuous regional arterial infusion; CT, Computed tomography; JCS, Japan Coma Scale.

Propensity score matching revealed that patient characteristics were similar in the adjusted CRAI and non-CRAI groups. In particular, the CT severity scores did not differ significantly between groups.

The protease inhibitors gabexate mesilate and nafamostat mesilate were administered to 60.4% and 39.6%, respectively, of the patients in the propensity-matched CRAI group, and to 58.9% and 41.1%, respectively, of the patients in the propensity-matched non-CRAI group. The antibiotics meropenem, imipenem and others were administered to 44.0%, 43.0% and 13.0%, respectively, of the patients in the propensity-matched CRAI group, and to 60.9%, 32.9% and 6.2%, respectively, of the patients in the propensity-matched non-CRAI group.

### Outcomes in the CRAI and non-CRAI groups

Among all 1,554 eligible patients, the in-hospital mortality rate was higher in the CRAI group than in the non-CRAI group (8.5% vs. 6.8%; *P* = 0.342). Outcomes in the propensity-matched CRAI and non-CRAI groups are summarized in Table 3. In-hospital mortality rates were similar (7.7% vs. 8.7%, odds ratio (OR) = 0.88, 95% CI = 0.44 to 1.78; *P* = 0.720). In-hospital mortality rates were 4.6% in patients who underwent CRAI on admission day 1, 10.0% on day 2 and 13.2% on day 3 or later. Earlier administration of CRAI tended to be associated with lower in-hospital morality rate (*P* = 0.064). In addition, length of hospital stay was significantly longer (*P* < 0.001) and total costs during hospitalization were significantly higher (*P* < 0.001) in the matched CRAI group than in the matched non-CRAI group, whereas the rate of interventions for infectious complications tended to be higher in the matched CRAI group (2.9% vs. 0.5%; *P* = 0.061). In detail, four patients in the matched CRAI group and one in the matched non-CRAI group required endoscopic necrosectomy, and two patients in the matched CRAI group required percutaneous drainage. No patient required surgical necrosectomy.

**Table 3:** **Outcomes in the propensity-matched continuous regional arterial infusion and non–continuous regional arterial infusion groups**^**a**^

**Outcomes**	**CRAI (*n*** **= 207)**	**Non-CRAI (*n*** **= 207)**	***P***
In-hospital mortality, *n *(%)	16 (7.7%)	18 (8.7%)	0.720
Median length of stay, days (IQR)	28.5 (18.3 to 36.8)	18.0 (12.0 to 28.0)	<0.001
Median cost, US$ (IQR)	$21,800 ($16,200 to $32,400)	$12,600 ($7,940 to $21,700)	<0.001
Interventions for infectious complications, *n *(%)	6 (2.9%)	1 (0.5%)	0.061

^a^CRAI, Continuous regional arterial infusion; IQR, Interquartile range.

Logistic regression analysis with adjustment for propensity score quintiles showed that the in-hospital mortality rate (adjusted OR = 0.88; 95% CI = 0.43 to 1.78; *P =* 0.711) and the rate of interventions for infectious complications (adjusted OR = 6.42, 95% CI = 0.75 to 54.6; *P =* 0.089) were similar in the matched CRAI and non-CRAI groups (Table 4). Linear regression analysis with adjustment for the same variables as those used in the calculation of propensity scores showed that hospital stay was 16.5 days longer (*P* < 0.001) and cost was US$13,600 higher (*P* < 0.001) in the matched CRAI cohort than in the matched non-CRAI group (Table 4).

**Table 4:** Odds ratios for in-hospital mortality and interventions for infectious complications and coefficients for length of stay and cost of the CRAI group, compared with the non-CRAI group

	**CRAI vs. non-CRAI**			
**Variable**	**Odds ratio**	**Coefficient**	**95% CI**	***P***
In-hospital mortality	0.88		0.43 to 1.78	0.711
Interventions for infectious complications	6.42		0.75 to 54.6	0.089
Length of stay (days)		16.5	11.8 to 21.2	<0.001
Cost (US dollars)		$13,600	6,890 to 20,400	<0.001

The odds ratios were calculated using logistic regression analysis with adjustment for propensity score quintiles, and the coefficients using linear regression analysis with adjustment for age, sex, JCS, CCI, prognostic factor score, CT score, hospital volume and hospital type.

## Discussion

To more accurately evaluate CRAI of a protease inhibitor and an antibiotic in patients with acute pancreatitis, we performed propensity score analysis based on large-scale data from the Japanese nationwide administrative database. We found that CRAI was ineffective in that it did not reduce the in-hospital mortality rate, but rather was associated with an increased rate of interventions for infectious complications associated with acute pancreatitis. In addition, CRAI was associated with significantly longer hospital stays and significantly higher total costs.

Several small retrospective studies have suggested that CRAI may be effective in patients with severe acute pancreatitis [11-15]. To date, only one randomized controlled trial has shown that CRAI reduced mortality rates, but that study was limited by its relatively small sample size (78 patients) [16]. CRAI was originally performed in patients with pancreatic necrosis caused by acute pancreatitis, a condition with a high mortality rate. This treatment selection bias has hindered a simple retrospective comparison evaluating the efficacy of CRAI; that is, patients who underwent CRAI were likely to be at greater risk of acute pancreatitis-associated mortality, thus leading to underestimation of the efficacy of CRAI. The younger age of our CRAI cohort relative to our non-CRAI group was likely to enhance outcomes in the former [30], whereas the higher prognostic factor and CT severity index scores in the CRAI group would likely have a negative effect on outcomes [31]. Thus, to control for baseline prognostic heterogeneity and inherent treatment selection biases, we performed propensity score analysis [27,28]. We found that one-to-one propensity score matching resulted in a successful balance of baseline characteristics, including CT severity score, the factor that usually determines whether CRAI is administered.

When we compared the propensity-matched groups, we found that, in contrast to previous studies, in-hospital mortality rates were similar between our CRAI and non-CRAI groups, despite the tendency of earlier administration of CRAI to be associated with a lower in-hospital mortality rate. Although we expected that CRAI of an antibiotic would result in a lower rate of infectious complications, we found that the rate of requirement for surgery and other interventions was higher in the CRAI group. In addition, hospital stay was significantly longer and total costs were significantly higher in the CRAI cohort than in the non-CRAI group.

This study has several limitations. First, it was not a prospective, randomized controlled trial, but rather was based on a retrospective design. Although we performed propensity score analysis to overcome this limitation, several biases may remain because of unobserved confounders. For example, the criteria for and timing of administration of CRAI were not predefined, and the changes in the prognostic factor and CT severity scores over time were unavailable from the DPC database. Thus, the condition of some patients diagnosed with “non-severe” acute pancreatitis upon admission may have worsened rapidly, thus unfavorably affecting the outcomes in our CRAI group. Second, CT severity score was determined by scoring and summing two independent factors: extrapancreatic progression of inflammation and hypoenhanced lesions of the pancreas. These two factors affect the efficacy of CRAI differently, although total CT severity scores were well-balanced in our adjusted CRAI and non-CRAI groups. Third, the unavailability of some important clinical data from the DPC database may have affected patient outcomes, including the etiologies of acute pancreatitis [32,33], symptoms of sepsis, data on organ failure, dosages of protease inhibitors and antibiotics on a daily basis and detailed results of blood tests and CT. The information on readmissions and outpatient visits was also unavailable, which inhibited the analysis of late mortality associated with acute pancreatitis. The multiplicity of protease inhibitors and antibiotics used constitutes another limitation.

Despite these limitations, a major strength of this study was the evaluation of CRAI for acute pancreatitis in a large number of patients with well-balanced baseline characteristics, including prognostic factor score affecting in-hospital mortality rate, such as age [30], sex [32,34], consciousness [30], prognostic factor score [31] and CT severity score. To the best of our knowledge, this study is the largest to date that has evaluated CRAI, and it included patients who underwent CRAI in a large number of hospitals (*n* = 99), including 70 nonacademic hospitals with a relatively low hospital volume and 29 academic hospitals, thus providing internal validity. Because of its invasiveness and cost, CRAI should not routinely be performed in patients with acute pancreatitis until its efficacy has been well-established and its indications are confirmed.

## Conclusions

On the basis of our propensity score analysis of large-scale data from a nationwide administrative database, we found that CRAI was not effective in the treatment of acute pancreatitis and that it cost more. Well-designed, randomized controlled trials including large numbers of patients are needed to further evaluate the efficacy of this procedure.

## Key messages

• Because little is known about the potential effectiveness of CRAI in patients with severe acute pancreatitis, the effectiveness and costs of CRAI were evaluated on the basis of data derived from a nationwide, large-scale database.

• One-to-one propensity score matching was performed to adjust for treatment selection bias, and 207 well-balanced pairs were compared.

• CRAI failed to demonstrate superiority regarding mortality rate, length of hospital stay and total cost, suggesting the need for large randomized controlled trials to further evaluate the effectiveness of CRAI.

## Abbreviations

CCI: Charlson comorbidity index; CI: Confidence interval; CRAI: Continuous regional arterial infusion; CT: Computed tomography; DPC: Diagnosis procedure combination; ICD-10: International classification of diseases and related health problems, 10th revision; IQR: Interquartile range; JCS: Japan coma scale; OR: Odds ratio.

## Competing interests

The authors declare that they have no competing interest.

## Authors’ contributions

TH participated in the study concept and design, analysis and interpretation of data, drafting of the manuscript and statistical analysis. HY was responsible for acquisition of data, study concept and design, analysis and interpretation of data, statistical analysis and critical revision of the manuscript for important intellectual content. YN and HI participated in the study concept and design, analysis and interpretation of data and critical revision of the manuscript for important intellectual content. HH, SM and KF were involved with acquisition of data and critical revision of the manuscript for important intellectual content. KK was responsible for study supervision and final approval of the manuscript. All authors read and approve the final manuscript.
